# Photoactivated iridium(III) complexes drive pyroptosis–necroptosis synergy for multi‐network photoimmunotherapy of renal cell carcinoma

**DOI:** 10.1002/smo2.70087

**Published:** 2026-07-28

**Authors:** Xin Qin, Meng‐Di Chen, Wenqi Gao, Yiping Wang, Lin‐Qing Liu, Zhen Teng, Tienan Qi, Qiyuan Wang, Yuxuan Wang, Daoxiang Li, Keqiang Yan, Ling Pan, Zhongwei Zhao, Shuo Zhao, Kang‐Nan Wang, Shuai Fu, Nengwang Yu

**Affiliations:** ^1^ Department of Urology Qilu Hospital of Shandong University Cheeloo College of Medicine Shandong University Jinan China; ^2^ State Key Laboratory of Crystal Materials Shandong University Jinan China; ^3^ Department of Radiation Oncology Qilu Hospital of Shandong University Cheeloo College of Medicine Shandong University Jinan China; ^4^ Department of Medical Oncology Shandong Cancer Hospital and Institute Shandong First Medical University and Shandong Academy of Medical Sciences Jinan China; ^5^ School of Biomedical Engineering Shandong University Jinan China; ^6^ Department of Urology Qilu Hospital of Shandong University Dezhou Hospital Dezhou China

**Keywords:** iridium complex, necroptosis, photodynamic therapy, pyroptosis, renal cell carcinoma

## Abstract

Recurrence and metastasis are the leading causes of mortality in renal cell carcinoma (RCC), and its intrinsic drug resistance further limits effective therapeutic options. Synergistic activation of multiple regulated cell death pathways has recently emerged as a novel approach to overcome therapeutic resistance. Here, we developed two mitochondria‐targeted iridium(III) photosensitizers, Ir‐MT1 and Ir‐MT2, for synergistic photoimmunotherapy of RCC. Upon white‐light irradiation, Ir‐MT1/2 induced severe mitochondrial damage and dysfunction, leading to massive release of mitochondrial contents. Mitochondrial DNA leakage activated the cGAS–stimulator of interferon genes pathway and caspase‐1‐mediated pyroptosis cascade, whereas excessive Ca^2+^ efflux promoted RIPK1/RIPK3 phosphorylation and induced necroptosis. These death signals facilitated pore formation by gasdermin D and mixed lineage kinase domain‐like protein in the plasma membrane, resulting in membrane rupture, release of damage‐associated molecular patterns, and immunogenic cell death synergistically. In vivo, Ir‐MT1/2 not only effectively suppressed primary tumor growth but also eliminated distant tumors through activation of anti‐tumor immunity, exhibiting potent therapeutic efficacy and favorable biosafety. Overall, our work provides the evidence that a single iridium complex can simultaneously trigger pyroptosis–necroptosis synergy, overcoming intrinsic drug resistance and offering a promising strategy for multi‐network systemic therapy of RCC.

## Graphical abstract

Photoactivated iridium(III) complexes trigger mitochondrial DNA leakage and Ca²⁺ efflux, simultaneously driving pyroptosis and necroptosis to synergistically enhance immunogenic cell death. This multi‐network cascade promotes dendritic cell maturation and CD8⁺ T cell activation, achieving potent photoimmunotherapy of renal cell carcinoma.

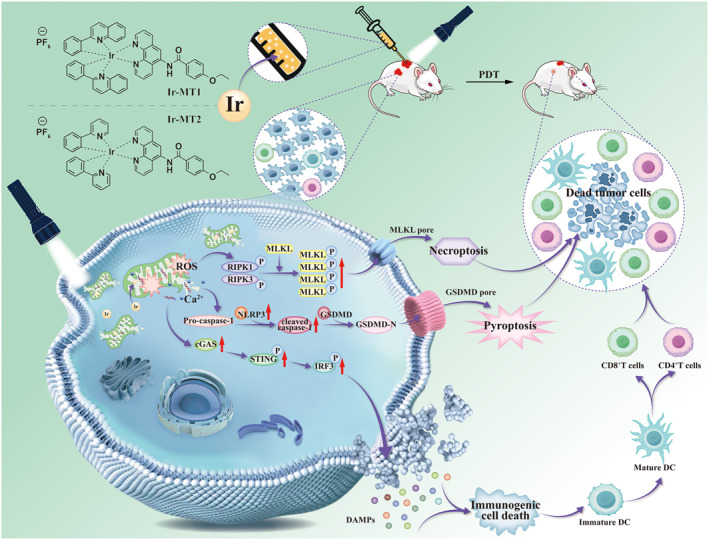

## 1 INTRODUCTION

Renal cell carcinoma (RCC) is one of the most common urological malignancies, and its recurrence and metastasis are the main causes of cancer‐related mortality.[^1^, ^2^] Unlike many other solid tumors, RCC is insensitive to cisplatin‐based chemotherapy and radiotherapy because of intrinsic apoptosis resistance and enhanced DNA repair, which pose major challenges for systemic treatment.[^3^, ^4^, ^5^] Currently, targeted therapy and immunotherapy constitute the main therapeutic options for advanced RCC. However, the rapid development of drug resistance and severe side effects greatly limit their clinical benefits for RCC patients.[^6^, ^7^, ^8^] Consequently, there is an urgent need to develop novel therapeutic strategies that can overcome drug resistance and initiate a lasting anti‐tumor immune response.

Photodynamic therapy (PDT) is a non‐invasive treatment modality that eliminates tumor cells by activating photosensitizers with light irradiation to generate cytotoxic reactive oxygen species (ROS).[^9^, ^10^, ^11^] Compared with conventional therapies, PDT offers the advantages of low systemic toxicity, high spatiotemporal accuracy and the ability to activate anti‐tumor immunity, making it a highly promising strategy for cancer treatment.[^12^, ^13^, ^14^] Nevertheless, the poor photostability, limited selectivity and short ROS lifetime of conventional photosensitizers severely compromise the therapeutic efficacy of PDT.[^15^, ^16^, ^17^] These drawbacks highlight the urgent need for novel photosensitizers to overcome the inherent limitations of PDT.

In recent years, iridium(III) complexes have gained considerable attention for their outstanding photophysical and biological properties, including long luminescence lifetimes, large Stokes shifts, high cellular uptake and superior photostability.[^18^, ^19^, ^20^] These characteristics make iridium(III) complexes promising candidates for next‐generation photosensitizers. However, most reported iridium(III) complexes mainly induce single‐form regulated cell death (RCD), such as apoptosis,[^21^, ^22^], autophagy,^[23]^ or other related pathways,[^24^, ^25^] which is often insufficient to overcome the intrinsic resistance of aggressive tumors.^[26]^ Notably, traditional apoptosis is generally recognized as an immunologically silent form of cell death and fails to effectively induce potent anti‐tumor immune responses,[^27^, ^28^] particularly within the highly immunosuppressive tumor microenvironment.^[29]^ This limitation is particularly pronounced in RCC, which is characterized by intrinsic resistance to apoptosis and robust immune evasion, thereby significantly reducing the therapeutic efficacy of conventional single‐pathway interventions.[^30^, ^31^, ^32^] In contrast, emerging immunogenic forms of RCD, such as pyroptosis and necroptosis, exhibit powerful immunostimulatory capacity.[^33^, ^34^] They are characterized by plasma membrane rupture and the release of damage‐associated molecular patterns (DAMPs), which can promote dendritic cell maturation and activate cytotoxic T lymphocyte‐mediated anti‐tumor immunity.[^35^, ^36^, ^37^] Importantly, both pyroptosis and necroptosis are non‐apoptotic programmed cell death pathways. This allows them to effectively avoid traditional apoptosis‐resistance networks and maintain cytotoxic efficacy even under conditions where classical apoptotic cascades are dysfunctional.[^38^, ^39^, ^40^] However, no photosensitizer has been reported that can simultaneously induce pyroptosis–necroptosis synergy to enhance photoimmunotherapy, and the underlying synergistic mechanisms remain poorly understood.

**SCHEME 1:**
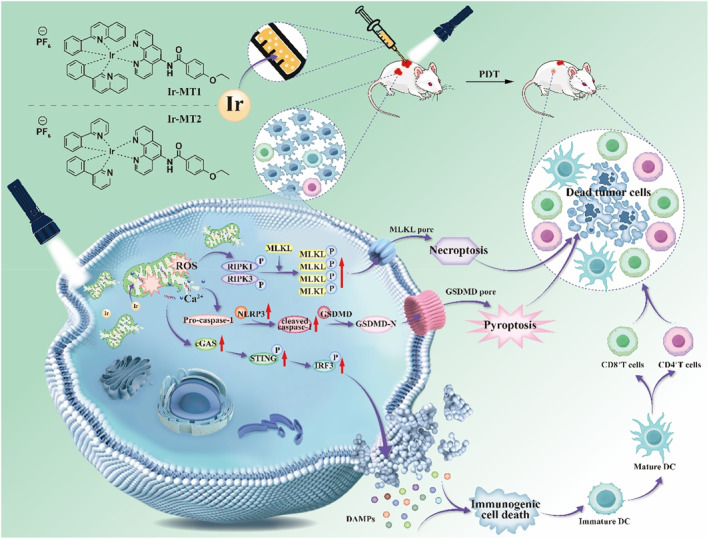
Chemical structures of Ir‐MT1/2 and the underlying mechanisms of pyroptosis–necroptosis synergy in multi‐network photoimmunotherapy of renal cell carcinoma.

In this study, we designed two novel mitochondria‐targeted iridium(III) photosensitizers (Ir‐MT1 and Ir‐MT2) to enhance the photodynamic immunotherapy of RCC (Scheme 1). Upon white‐light irradiation, Ir‐MT1/2 could efficiently generate ROS, leading to severe mitochondrial damage and dysfunction, which in turn induced massive release of mitochondrial contents. Notably, leakage of mitochondrial DNA (mtDNA) activated the cGAS–stimulator of interferon genes (STING) pathway and caspase‐1‐mediated pyroptosis cascade, whereas excessive Ca^2+^ efflux promoted the phosphorylation of RIPK1/RIPK3 and induced necroptosis. These death signals drove gasdermin D (GSDMD)‐ and mixed lineage kinase domain‐like protein (MLKL)‐mediated membrane pore formation, leading to membrane rupture, release of DAMP, and synergistic induction of immunogenic cell death (ICD). In vivo, Ir‐MT1/2 significantly promoted the maturation of DCs and activation of CD8^+^ T cells, exhibiting pronounced anti‐tumor effects and robust immunogenicity. In summary, our study provides the evidence that a single iridium complex can simultaneously induce pyroptosis and necroptosis to achieve efficient tumor eradication and potent immune response, overcoming intrinsic drug resistance and offering a promising strategy for multi‐network synergistic therapy of RCC.

## 2 METHODS

### 2.1 Synthesis of Ir‐complexes

The ligand 4‐ethoxy‐N‐(1,10‐phenanthrolin‐5‐yl) benzamide (L) was synthesized via an amide condensation reaction. 1,10‐phenanthrolin‐5‐amine (0.19 g, 1.0 mmol) and 4‐ethoxybenzoyl chloride (0.18 g, 1.0 mmol) were dissolved in anhydrous dichloromethane (30 mL). To this solution, triethylamine (0.10 g, 1.0 mmol) was added dropwise. The reaction mixture was stirred at room temperature for 24 h and then evaporated to dryness. The residue was purified by silica gel column chromatography (eluent: ethyl acetate/petroleum ether, 4:1 v/v) to afford L as a grayish‐white solid (0.25 g, yield 75.8%). Synthesis of μ‐dichloro‐bridged dimers: Iridium (III) chloride trihydrate (0.30 g, 1.0 mmol) reacted with 2‐phenylquinoline (for phq) (0.51 g, 2.5 mmol) and 2‐phenylpyridine (for ppy) (0.39 g, 2.5 mmol), respectively, in a mixture of 2‐ethoxyethanol and water (3:1, v/v, 50 mL) under reflux for 24 h. The reaction mixtures were stirred at room temperature for 24 h and then evaporated to dryness. The residue was purified by silica gel column chromatography to form the μ‐dichloro‐bridged dimers [Ir(phq)2(μ‐Cl)]2 (0.70 g, yield 55.1%) and [Ir(ppy)2(μ‐Cl)]2 (0.61 g, yield 56.7%). Synthesis of Ir‐MT1/2: a mixture of dimer [Ir(phq)2(μ‐Cl)]2 (for Ir‐MT1) (0.13 g, 0.1 mmol) or [Ir(ppy)2(μ‐Cl)]2 (for Ir‐MT2) (0.11 g, 0.1 mmol) and ligand L (0.075 g, 0.21 mmol) was refluxed in a CH_2_Cl_2_/CH_3_OH (1:1, v/v) solvent mixture for 6 h. The reaction was terminated by 0.4 mmol NH_4_PF_6_ and then subjected to evaporation until complete dryness. The residue was purified by silica gel column chromatography (eluent: CH_2_Cl_2_/CH_3_OH, 10:1, v/v) to yield Ir‐MT1 and Ir‐MT2 as yellow‐green solids in 52.3% and 52.6% yields, respectively.

### 2.2 UV‐vis spectra and fluorescence spectra assay

Stock solutions of Ir‐MT1 and Ir‐MT2 were prepared in dimethyl sulfoxide (DMSO) at a concentration of 1 mM each. These stock solutions were then diluted in 1,4‐dioxane, DMSO, and H_2_O to a final concentration of 10 μM for spectral measurements. UV‐Vis absorption spectra were obtained using full‐wavelength scanning on a Hitachi U2910 spectrophotometer at 298 K. Subsequently, fluorescence emission spectra were recorded from 415 to 800 nm with an excitation wavelength of 405 nm. The relative fluorescence quantum yields were determined with [Ru(bpy)3]^2+^ as the standard. Fluorescence lifetime and steady state were measured by an FLS‐980 fluorescence spectrometer (Edinburgh Instruments).

### 2.3 pH‐responsive behavior

Stock solutions of Ir‐MT1 and Ir‐MT2 were prepared in H_2_O at a concentration of 10 μM. The pH of these solutions was then adjusted across a range from 3.0 to 8.0 using Britton‐Robinson buffer. Fluorescence emission spectra of the pH‐adjusted samples were recorded upon excitation at 405 nm. The relative fluorescence intensity was calculated as the ratio of the emission maximum at 564 nm at each pH to that in H_2_O.

### 2.4 Ions/bioactive molecular‐response assay

Stock solutions of Ir‐MT1 and Ir‐MT2 were prepared in H_2_O at a concentration of 10 μM, and then were added different ions/bioactive molecular with a final concentration of 200 μM, including control, HSO_3_^−^, Cl^−^, SO_4_^2−^, NO_3_^−^, HS^−^, CO_3_^2−^, I^−^, Ag^+^, K^+^, Ca^2+^, Mg^2+^, Na^+^, Al^3+^, Cu^2+^, Hg^2+^, Fe^3+^, Zn^2+^, glutathione and Cys. The solutions were detected, and the relative fluorescence intensity was calculated as aforementioned.

### 2.5 Detection of ROS generation

The ability of Ir‐MT1 and Ir‐MT2 to generate ROS was evaluated using specific fluorescent probes: 9,10‐anthracenediyl‐bis(methylene)dimalonic acid (ABDA) for singlet oxygen (^1^O_2_), hydroxyphenyl fluorescein (HPF) for hydroxyl radical (·OH), DHR123 for superoxide anion (O_2_^−^·), and 2′,7′‐dichlorofluorescein (DCF) as a total ROS indicator. To achieve an initial absorbance close to 1.0 at the relevant wavelength, the probe test concentrations of HPF, DHR123, and DCF were 10 μM, while that of ABDA was 100 μM.

Experimentally, a 10 μM solution of Ir‐MT1 or Ir‐MT2 was mixed evenly with each indicator probe. The mixture was then transferred to a 1‐cm path length quartz cuvette and irradiated with a white LED light source (30 mW/cm^2^) for specified time intervals. Following irradiation, the absorption or fluorescence emission spectra were recorded immediately. A control experiment, containing the indicator solution without the Ir complexes, was performed in parallel. The ROS‐generating ability was evaluated by analyzing the time‐dependent spectral changes of each probe relative to its control. Flow cytometric analysis was performed on a BD FACS Celesta Multicolor Flow Cytometer, and data were analyzed using FlowJo software.

### 2.6 Lipophilicity measurement

Equal volumes (2 mL each) of n‐octanol and water were combined and shaken at constant temperature for 24 h to achieve mutual saturation. The mixture was then allowed to stand to achieve complete phase separation. Ir‐MT1 or Ir‐MT2 was added to the pre‐saturated biphasic system at 10 μM. This mixture was equilibrated by shaking overnight at constant temperature. After phase separation, the concentration of the complex in each layer was quantified by UV‐Vis spectroscopy. Log P_o_/_w_ was derived from the organic‐to‐aqueous phase concentration ratio.

### 2.7 Cell lines and cell culture

Human‐derived RCC cell lines (786‐O and A498), mouse‐derived RCC cell lines (Renca) and human renal tubular epithelial cell lines (HK‐2) were used in the present study. Renca was purchased from American Type Culture Collection, and other cell lines were purchased from Shanghai Institute of Biochemistry and Cell Biology Cell Bank. Renca was cultured in RPMI‐1640 medium (Thermo Fisher Scientific) with 10% fetal bovine serum (FBS), 0.1 mM non‐essential amino acids, 1 mM Sodium Pyruvate, and 2 mM L‐glutamine (Thermo Fisher Scientific). 786‐O was cultured in RPMI‐1640 medium with 10% FBS. A498 and HK‐2 were cultured in Dulbecco’s modified Eagle’s medium (Thermo Fisher Scientific) with 10% FBS. All cell lines were incubated at 37°C in a humidified atmosphere with 5% CO2 and tested negative for *Mycoplasma* bacteria.

### 2.8 Cell viability analyses

786‐O cells (10,000 per well) were seeded in 96‐well plates. Each well was treated with specific concentrations of Ir‐MT1/2 for 2h and followed by white‐light irradiation for 5 min. Cell death inhibitors (MedChemExpress) were added with 0.5 μM Ir‐MT1/2 for further analyses. Cell viability was measured using CCK‐8 assays (Dojindo).

### 2.9 Cell imaging

786‐O cells were plated in confocal dishes. For colocalization analyses, 786‐O cells were treated with 5 μM Ir‐MT1/2 for 2 h, followed by staining with MitoTracker Deep Red (Beyotime) for 15 min. For phenotype analyses, 786‐O cells were treated with 0.5 μM Ir‐MT1/2 for 2h and followed by white‐light irradiation for 5 min. ROS generation, intracellular Ca^2+^ levels, and mitochondrial membrane potential were assessed using H2DCFDA (MedChemExpress, 10 μM, 30 min), Fluo‐4 AM (YEASEN, 20 μM, 30 min), and JC‐1 (Beyotime, 10 μg/mL, 20 min) respectively. mtDNA was traced with PicoGreen (YEASEN, 1:200 dilution) for 30 min.^[41]^ The cell samples were detected using an Olympus SpinSR10 spinning disk confocal super‐resolution microscope and the Opera Phenix® Plus High Content Screening System (PerkinElmer) in confocal mode.

### 2.10 RT‐qPCR

To detect cytosolic mtDNA, treated 786‐O cells were incubated with 1% NP‐40 on ice (Beyotime) for 15 min. The supernatants were collected and centrifuged at 16,215 × *g* for 15 min at 4°C. mtDNA was then extracted using the TIANamp kit (TIANGEN). Relative mtDNA levels were quantified on a CFX Connect Real‐Time polymerase chain reaction System (Bio‐Rad) with SYBR Green reagents (Accurate Biology). The primers are listed in Table S3.

### 2.11 Western blot

Total proteins from treated cells were extracted using 1× radioimmunoprecipitation assay buffer lysis buffer (Beyotime) supplemented with a protease and phosphatase inhibitor cocktail (Beyotime). The cell lysates were incubated on ice for 30 min and then clarified by high‐speed centrifugation at 12,000 rpm for 15 min at 4°C. Protein concentration was determined using a bicinchoninic acid Protein Assay Kit (Beyotime). Protein was mixed with 5× sodium dodecyl sulfate (SDS) loading buffer and denatured at 95°C for 5 min. 20 μg protein were separated by SDS–polyacrylamide gel electrophoresis and subsequently transferred onto polyvinylidene fluoride membranes (Millipore) using a wet transfer system at 110 V for 90 min under ice‐cold conditions. Membranes were blocked with 5% bovine serum albumin in Tris‐buffered saline with Tween‐20 for 1 h at room temperature. The membranes were incubated with primary antibodies overnight at 4°C, followed by incubation with secondary antibodies for 1 h at room temperature. Protein bands were visualized using Super enhanced chemiluminescence Detection Reagent (YEASEN). All antibodies used in western blot, including the catalog numbers and dilution ratios, are summarized in detail in Table S4.

### 2.12 Immunohistochemistry

Tissue sections were deparaffinized in xylene and rehydrated through a graded ethanol series (100%, 95%, 85%, and 75%) in distilled water. Sections were heated in EDTA buffer (pH 8.0, Beyotime) using a microwave for 15 min, followed by natural cooling to room temperature. Endogenous peroxidase activity was inhibited by incubation with H_2_O_2_ for 15 min at room temperature. Non‐specific binding sites were blocked using 10% goat serum for 30 min at room temperature. The sections were incubated with primary antibodies overnight at 4°C. After washing with phosphate‐buffered saline, the tissue sections were then incubated with secondary antibodies for 1 h at room temperature. The labeled proteins were visualized using 3,3′‐diaminobenzidine staining (Thermo Fisher Scientific), and images were captured using the Olympus VS200 microscope. All antibodies used in immunohistochemistry, including the catalog numbers and dilution ratios, are summarized in detail in Table S4.

### 2.13 Animal experiments

One million Renca cells were initially inoculated into the right flank of immunocompetent BALB/c mice (Beijing Vital River Laboratory Animal Technology) to establish primary tumors. Five days later, one million Renca cells were inoculated into the left flank to serve as distant tumors. Ir‐MT1/2 (10 mg/kg) was intratumorally injected and followed by white‐light irradiation on the second day after injection. This process was repeated four times, with tumor volume and body weight measured every 3 days.

### 2.14 Statistical analyses

Differences between two groups were compared using Student's *t*‐test, with independent *t*‐tests applied for normally distributed data and the Mann–Whitney *U* test used for non‐normally distributed data. One‐way analysis of variance was used for comparisons among multiple groups. Spearman's correlation coefficient was calculated to assess the relationship between two continuous variables. All analyses with a *p*‐value of <0.05 was considered statistically significant.

## 3 RESULTS AND DISCUSSIONS

### 3.1 Preparation and characterization of Ir‐MTs

**FIGURE 1:**
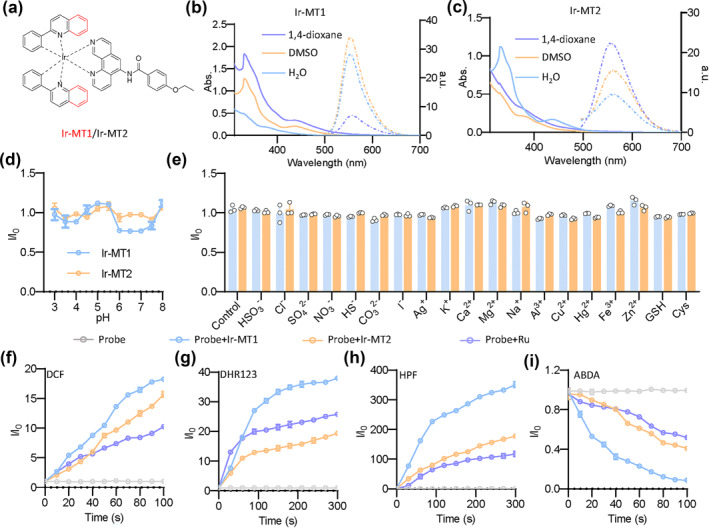
Photophysical characterized of Ir‐MT1/2. (a) Structure of Ir‐MT1/MT2. (b) UV‐vis spectra and emission fluorescence of IrMT1 in 1,4‐dioxane, DMSO and H_2_O at 298K (*λ*_ex_ = 405 nm). (c) UV‐vis spectra and emission fluorescence of IrMT2 in 1,4‐dioxane, DMSO and H_2_O at 298K (*λ*_ex_ = 405 nm). (d) pH‐response of Ir‐MT1/2 in different buffer range buffer from pH 3.0 to pH 8.0. (e) Stability of Ir‐MT1/2 in different ions and bioactive molecular with 200 μM. (f) Reactive oxygen species levels of Ir‐MT1/2 was detected via DCF. (g) Superoxide radical (O_2_^−^) of Ir‐MT1/2 was detected via DHR123. (h) Hydroxyl radicals (•OH) levels of Ir‐MT1/2 were detected via HPF. (i) Singlet oxygen (^1^O_2_) levels of Ir‐MT1/2 were detected via ABDA. ABDA, 9,10‐anthracenediyl‐bis(methylene)dimalonic acid; DCF, 2′,7′‐dichlorofluorescein; DMSO, dimethyl sulfoxide; HPF, hydroxyphenyl fluorescein.

The synthesis pathways of Ir‐complexes are shown in Scheme S1. Firstly, the ligand 4‐ethoxy‐N‐(1,10‐phenanthrolin‐5‐yl) benzamide (termed L) was synthesized from 1,10‐phenanthrolin‐5‐amine and 4‐ethoxybenzoyl chloride via amide condensation reaction. Next, the μ‐dichloro‐bridged dimer [Ir(phq)_2_(μ‐Cl)]_2_ and [Ir(ppy)_2_(μ‐Cl)]_2_ was synthesized from 2‐phenylquinoline (for phq) and 2‐phenylpyridine (for ppy), respectively, with iridium (III) chloride trihydrate in 2‐ethoxyethanol under reflux. Finally, treatment of the dimer precursors [Ir(phq)_2_(μ‐Cl)]_2_ and [Ir(ppy)_2_(μ‐Cl)]_2_ with ligand L in refluxing CH_2_Cl_2_/CH_3_OH (1:1, v/v) afforded, after purification by silica gel column chromatography, the target complexes Ir‐MT1 and Ir‐MT2, respectively (Figure 1a). To investigate the electronic absorption spectra, Ir‐MT1 and Ir‐MT2 were dissolved in H_2_O, DMSO and 1,4‐dioxane for assays. Results showed that both Ir‐MT1 and Ir‐MT2 showed strong absorption below 350 nm, primarily due to the spin‐allowed π‐π* transitions of the free ligands; broad bands at 350–450 nm from ligand‐centered charge transfers; and absorptions beyond 400 nm assigned to both singlet and triplet metal‐to‐ligand charge transfer transitions, with the latter enhanced by spin‐orbit coupling (Figure 1b,c). Upon excitation at 405 nm, the emission spectra of Ir‐MT1 and Ir‐MT2 both exhibited a single maximum at approximately 560 nm, with no distinct spectral shift observed (Figure 1b,c). Their emission intensities and lifetimes were solvent‐dependent. The order for Ir‐MT1 was DMSO > H_2_O > 1,4‐dioxane, while for Ir‐MT2, it was H_2_O > DMSO >1,4‐dioxane. The relevant photophysical data are summarized in Table S1. Besides, the stability of Ir‐MT1 and Ir‐MT2 under different pH conditions was also examined to evaluate their adaptability to intracellular environments (Figure 1d). Spectral measurements revealed that Ir‐MT1 possesses better stability than Ir‐MT2. Furthermore, the emission spectra of Ir‐MT1 and Ir‐MT2 were measured in the presence of various ions and bioactive agents (each at 200 μM). Both complexes exhibited normal luminescence, indicating that their emission is not quenched by common intracellular inorganic salts and compounds, which demonstrates their good stability in such environments (Figure 1e). The measured logP values (a metric of lipophilicity) for Ir‐MT1 and Ir‐MT2 were 1.84 and 2.30, respectively (Table S1), confirming their lipophilic character and suggesting potential for cellular uptake.

Coordination‐metalated iridium complexes generate cytotoxic singlet oxygen efficiently via their long‐lived triplet states, which underlies their anti‐tumor activity in PDT. To evaluate the free radical generation capacity of the Ir‐complexes, their performance was compared with that of the common PDT agent [Ru(bpy)_3_]^2+^ (Ru) under white light irradiation using specific fluorescent probes. The results showed that the order of free radical/ROS generation detected by the DCF probe is Ir‐MT1 > Ir‐MT2 > Ru (Figure 1f). Quantitatively, the O_2_^−^ scavenging capacity of Ir‐MT1, as detected by the DHR123 probe, was approximately 1.48‐fold that of Ru. In contrast, Ir‐MT2 showed a reduced capacity (Figure 1g). Meanwhile, the ·OH generation capacities of Ir‐MT1 and Ir‐MT2 were 2.96‐ and 1.63‐fold higher, respectively (Figure 1h). ABDA probe detection showed that Ir‐M1 generated ^1^O_2_ with 2.50‐ and 1.83‐fold higher efficiency than Ir‐M2 and Ru, respectively (Figure 1i). These suggest that both Ir‐complexes potentiate PDT via the generation of various ROS.

### 3.2 Potent photocytotoxicity of Ir‐MT1/2 in RCC

**FIGURE 2:**
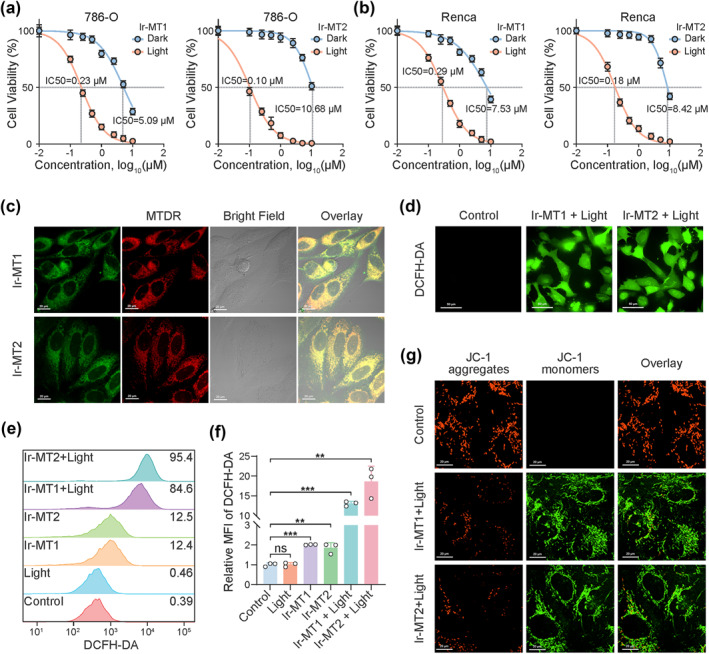
Ir‐MT1/2 exhibited photocytotoxicity through ROS generation. (a) Effects of Ir‐MT1 and Ir‐MT2 on the proliferation and viability of 786‐O cells, with six replicates per group. (b) Effects of Ir‐MT1 and Ir‐MT2 on the proliferation and viability of Renca cells, with six replicates per group. (c) Colocalization of Ir‐MT1/2 with MitoTrack Deep Red (MTDR) was detected by confocal microscopy in 786‐O cells. (d) Intracellular ROS levels in 786‐O cells were visualized by confocal microscopy using DCFH‐DA staining. (e) Intracellular ROS levels in 786‐O cells were analyzed by flow cytometry using DCFH‐DA staining. (f) The mean fluorescence intensity of DCFH‐DA was quantified to evaluate intracellular ROS levels. Three replicates were prepared per group. (g) Mitochondrial membrane potential in 786‐O cells was visualized by confocal microscopy using JC‐1 staining. DCFH‐DA, 2′,7′‐dichlorodihydrofluorescein diacetate; ROS, reactive oxygen species.

Although cisplatin is a widely used chemotherapeutic agent, its clinical application in RCC is limited by the intrinsic chemoresistance of RCC cells and severe nephrotoxicity caused by renal tubular injury.^[42]^ To systematically compare the cytotoxic profiles of cisplatin and Ir‐MT1/2, we evaluated their antiproliferative effects in human‐derived RCC cells (786‐O and A498), mouse‐derived RCC cells (Renca) and human renal tubular epithelial cells (HK‐2) using the CCK‐8 assay. As summarized in Table S2, cisplatin exhibited IC_50_ values exceeding 37 μM in all tested cell lines, with markedly higher cytotoxicity in HK‐2 than in RCC cells, and showed no significant difference between light and dark conditions. In contrast, Ir‐MT1/2 demonstrated pronounced photocytotoxicity in RCC under white‐light irradiation. Ir‐MT2 exhibited IC_50_ values of 0.11–0.17 μM and propidium iodide (PI) values of 50–97.45, whereas Ir‐MT1 showed slightly lower efficacy (IC_50_: 0.24–0.34 μM; PI: 18.29–36.45), yet both were markedly more effective than cisplatin (Figure 2a,b, Table S2). Meanwhile, no significant difference in tumor growth was observed between the white‐light irradiation‐only group and the control group (Supplementary Figure S1). Notably, unlike cisplatin, both Ir‐MT1 and Ir‐MT2 exhibited lower photocytotoxicity toward HK‐2 than RCC cells, indicating favorable selectivity and biocompatibility (Table S2). Collectively, these findings highlight the potent phototherapeutic potential of Ir‐MT1/2, particularly Ir‐MT2, and suggest that they may exert their effects through mechanisms distinct from cisplatin, warranting further investigation into their biological functions and potential clinical applications in RCC.

### 3.3 Efficient ROS generation and severe mitochondrial damage induced by Ir‐MT1/2

**FIGURE 3:**
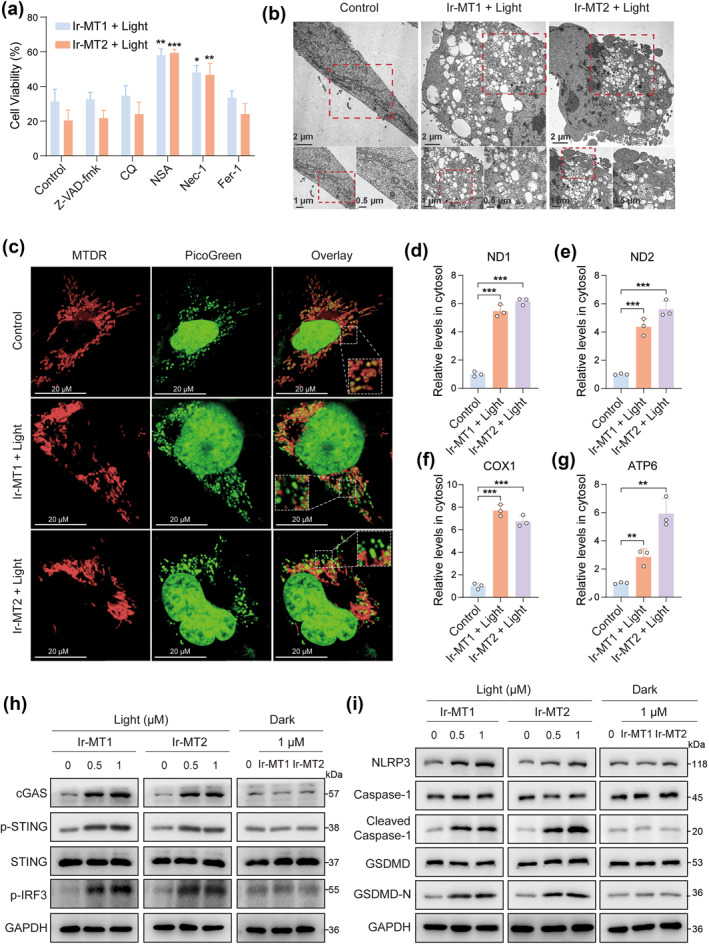
Ir‐MT1/2 triggers pyroptosis through mtDNA release. (a) Effects of a panel of cell death inhibitors on Ir‐MT1/2‐induced phototoxicity, with six replicates per group. (b) Morphological changes of 786‐O cells induced by Ir‐MT1/2 were observed through transmission electron microscopy. (c) Intracellular distribution of mtDNA in 786‐O cells was visualized by confocal microscopy using PicoGreen staining. PicoGreen: double‐stranded DNA; MitoTrack Deep Red: mitochondria. (d–g) Relative levels of mitochondria‐encoded genes leaked to the cytoplasm were measured by RT‐qPCR, with three replicates per group. (h) Western blot analysis of cGAS, p‐STING, STING and p‐IRF3 protein levels in 786‐O cells treated with Ir‐MT1/2. (i) Western blot analysis of NLRP3, Caspase‐1, cleaved Caspase‐1, GSDMD and GSDMD‐N protein levels in 786‐O cells treated with Ir‐MT1/2. GSDMD, gasdermin D; mtDNA, mitochondrial DNA; STING, stimulator of interferon genes.

To further elucidate the cellular mechanisms of Ir‐MT1/2, we examined their intracellular distribution using confocal microscopy. Both Ir‐MT1 and Ir‐MT2 colocalized excellently with the mitochondria‐specific probe MitoTracker Deep Red (MTDR). Pearson's correlation coefficients were 0.87 for Ir‐MT1 and 0.89 for Ir‐MT2, indicating strong mitochondrial targeting capability (Figure 2c). We next assessed their intracellular ROS‐generating ability in RCC cells using 2′,7′‐dichlorodihydrofluorescein diacetate. Treatment with 0.5 μM Ir‐MT1 or Ir‐MT2 followed by white‐light irradiation induced a significant increase in green fluorescence intensity compared with the control (Figure 2d). Flow cytometry analysis revealed that intracellular ROS levels in 786‐O cells increased by approximately 13‐ and 18.6‐fold respectively (Figure 2e,f, Supplementary Figure S2). This high ROS‐generating efficiency was consistent with the extracellular experimental results. Given that mitochondria are the central hub of cellular energy metabolism and signaling in tumor cells, excessive ROS accumulation can disrupt mitochondrial membrane potential and permeability, leading to mitochondrial damage and dysfunction.^[43]^ The mitochondria membrane potential was determined by JC‐1 staining. In 786‐O cells treated with Ir‐MT1/2, the ratio of red to green JC‐1 fluorescence significantly decreased after white‐light irradiation, indicating a loss of mitochondrial membrane potential (Figure 2g, Supplementary Figure S3). Furthermore, transmission electron microscopy (TEM) revealed pronounced mitochondrial swelling and vacuolization (Figure 3b). Taken together, these findings demonstrate that Ir‐MT1/2 induces severe mitochondrial damage.

### 3.4 Ir‐MT1/2 trigger cGAS‐STING signaling and pyroptosis cascade through mitochondrial DNA release

A panel of cell death inhibitors was employed to explore the mechanism underlying Ir‐MT1/2‐induced cell death. While PDT typically induces apoptosis, treatment with Z‐VAD‐FMK (an apoptosis inhibitor), chloroquine (an autophagy inhibitor), or Fer‐1 (a ferroptosis inhibitor) did not attenuate the cytotoxic effects of Ir‐MT1/2 (Figure 3a). In contrast, necrosulfonamide (NSA) (a pyroptosis inhibitor) and Nec‐1 (a necroptosis inhibitor) markedly improved cell viability, with NSA exhibiting a more pronounced protective effect than Nec‐1 (Figure 3a). These results suggest that Ir‐MT1/2 may induce both pyroptosis and necroptosis upon white‐light irradiation. To further investigate the mode of cell death, the morphological alterations were examined by TEM. Following Ir‐MT1/2 treatment combined with white‐light irradiation, 786‐O cells exhibited pronounced cellular and mitochondrial swelling, extensive intracellular vesiculation, plasma membrane rupture, cytoplasmic content release, and the formation of pyroptotic bodies (Figure 3b, Supplementary Figure S4). These morphological characteristics are consistent with both pyroptosis and necroptosis. Mitochondrial swelling and rupture can lead to the release of mitochondrial constituents, and recent studies have demonstrated that leakage of mtDNA into the cytosol activates the cGAS‐STING inflammatory signaling pathway, markedly amplifying the inflammatory response and promoting pyroptosis.[^44^, ^45^] Compared with the control group, PicoGreen staining of double‐stranded DNA (dsDNA) revealed a massive leakage of mtDNA signals into the cytosol following irradiation (Figure 3c). Consistently, RT‐qPCR revealed a significant enrichment of mitochondrial‐encoded genes in the cytosolic fraction (Figure 3d–g). Western blot analysis further showed elevated cGAS expression and increased phosphorylation of STING and IRF3, confirming activation of the cGAS‐STING pathway (Figure 3h). Upregulation of cGAS, a key cytosolic dsDNA sensor, further corroborates mtDNA release from mitochondria. Meanwhile, irradiation in the presence of Ir‐MT1/2 markedly increased NLRP3 expression and elevated the levels of cleaved caspase‐1 and GSDMD‐N, indicating inflammasome activation and formation of GSDMD‐mediated pyroptotic pores. These findings demonstrate that Ir‐MT1/2 induces pyroptosis through the canonical caspase‐1‐mediated pathway (Figure 3i).

### 3.5 Ir‐MT1/2 activate RIPK1/RIPK3‐mediated necroptosis via mitochondrial Ca^2+^ dysregulation

**FIGURE 4:**
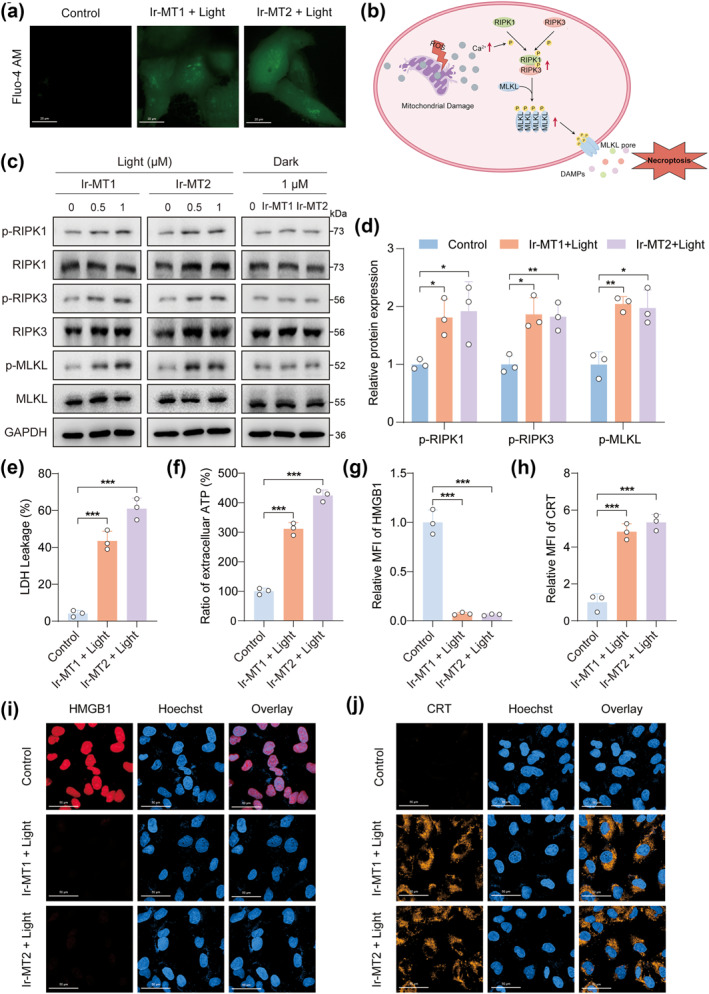
Ir‐MT1/2 trigger necroptosis and ICD. (a) Intracellular Ca^2+^ levels in 786‐O cells were visualized by confocal microscopy using Fluo‐4 AM staining. (b) Cytoplasmic calcium overload induces necroptosis by promoting the phosphorylation of RIP1 and RIP3. (c) Western blot analysis of RIPK1, RIPK3 and MLKL phosphorylation levels in 786‐O cells treated with Ir‐MT1/2. (d) The relative gray value of p‐RIPK1, p‐RIPK3 and p‐MLKL, with three replicates per group. (e) LDH leakage assay for 786‐O cells treated with Ir‐MT1/2. (f) Relative adenosine triphosphate levels in the culture supernatants of 786‐O cells, with three replicates per group. (g, h) The mean fluorescence intensity of HMGB1 (g) and calreticulin (h) in 786‐O cells treated with Ir‐MT1/2. Three replicates were prepared per group. (i, j) HMGB1 (i) and CRT (h) levels in 786‐O cells were visualized by confocal microscopy. AM, acetoxymethyl ester; HMGB1, high‐mobility group box 1; ICD, immunogenic cell death; LDH, lactate dehydrogenase; MLKL, mixed lineage kinase domain‐like protein.

Mitochondria play a crucial role in maintaining intracellular Ca^2+^ homeostasis, and their damage leads to a rapid Ca^2+^ efflux and dysregulation.^[46]^ Fluo‐4 acetoxymethyl ester, a fluorescent probe for free Ca^2+^, was used to monitor intracellular Ca^2+^ changes. The result of confocal microscopy revealed that treatment with Ir‐MT1/2 following irradiation significantly increased green fluorescence intensity in the cytoplasm, indicating elevated intracellular Ca^2+^ levels (Figure 4a). Cytoplasmic calcium overload is one of the most common causes of necroptosis in tumor cells, as it promotes the cascade phosphorylation of RIP1/RIP3/MLKL, leading to classical necroptosis (Figure 4b).^[47]^ Western blot analysis showed that while the expression of RIPK1, RIPK3, and MLKL remained unchanged after Ir‐MT1/2 treatment, their phosphorylation levels increased in a dose‐dependent manner (Figure 4C–D). To assess plasma membrane permeabilization, we measured lactate dehydrogenase (LDH) leakage. Figure 4E shows that compared to the control group, LDH release increased by 10.6‐ and 14.9‐fold in the Ir‐MT1 and Ir‐MT2 treatment groups, respectively, indicating severe membrane damage. Altogether, these findings suggest that Ir‐MT1/2 induces tumor cell death through RIPK1/RIPK3‐mediated necroptosis.

### 3.6 Ir‐MT1/2 induce ICD in vitro

Both pyroptosis and necroptosis increase plasma membrane permeability and pore formation, triggering ICD and the release of DAMPs, which include surface exposure of calreticulin (CRT), release of high‐mobility group box 1 (HMGB1), and extracellular secretion of adenosine triphosphate (ATP). The release of DAMPs promotes antigen exposure, activating long‐lasting anti‐tumor immunity.^[48]^ Therefore, we next investigated the ability of Ir‐MT1/2 to induce ICD in 786‐O cells. As shown in Figure 4g,i, immunofluorescence imaging revealed that treatment with Ir‐MT1/2 and white‐light irradiation significantly decreased intracellular red fluorescence of HMGB1. Meanwhile, white‐light irradiation induced a marked increase in orange fluorescence of CRT on the cell surface, indicating enhanced CRT translocation and membrane exposure (Figure 4h,j). Additionally, Ir‐MT1/2 treatment resulted in significantly higher ATP release compared to the control group (Figure 4f). These results demonstrate that Ir‐MT1/2 can induce ICD by promoting the release of DAMP, thereby enhancing the immunogenicity of RCC cells.

### 3.7 Ir‐MT1/2 exhibit potent antitumor efficacy and enhance antitumor immunity in vivo

**FIGURE 5:**
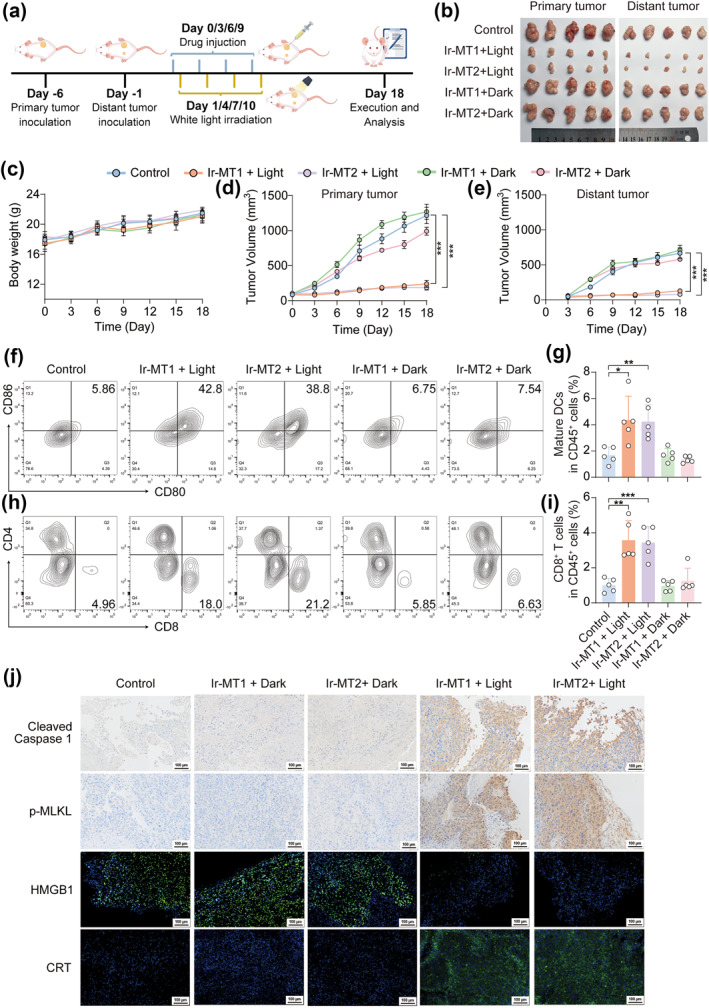
Ir‐MT1/2 exhibits potent anti‐tumor efficacy and enhance anti‐tumor immunity in vivo. (a) Schematic illustration of the in vivo experimental workflow. (b) Representative images of Ir‐MT1/2 treatment on bilateral tumor burden in BALB/c mice. (c) Changes in body weight of BALB/c mice during Ir‐MT1/2 treatment. (d) Effects of Ir‐MT1/2 treatment on primary tumor burden in BALB/c mice. (e) Effects of Ir‐MT1/2 treatment on distant tumor burden in BALB/c mice. (f, g) Changes in the abundance of CD80^+^CD86^+^ DCs after Ir‐MT1/2 treatment. (h, i) Changes in the abundance of CD8^+^ T cells after Ir‐MT1/2 treatment. (j) Immunohistochemical images of cleaved Caspase‐1, p‐MLKL, high‐mobility group box 1 and calreticulin in tumor tissues after Ir‐MT1/2 treatment.

Given the potent tumor‐killing effects and ICD induction ability of Ir‐MT1/2 in vitro, we further evaluated their anti‐tumor efficacy and immune activation potential in vivo. As shown in Figure 5a, Renca cells were initially inoculated into the right flank of immunocompetent BALB/c mice to establish primary tumors. Five days later, Renca cells were inoculated into the left flank to serve as distant tumors. Ir‐MT1 and Ir‐MT2 were intratumorally injected into the corresponding primary tumors, followed by white‐light irradiation on the second day after injection. This process was repeated four times, with tumor volume and body weight measured every 3 days. The results showed that Ir‐MT1/2 treatment combined with white‐light irradiation significantly suppressed primary tumor growth compared to the control group (Figure 5b,d). Importantly, no significant change in body weight was observed in the mice, indicating that the treatment was well tolerated (Figure 5c). Hematoxylin and eosin staining revealed no apparent histological changes in the major organs of the mice, further suggesting the good biocompatibility of Ir‐MT1/2 (Supplementary Figure S5). Moreover, the distant tumors in the Ir‐MT1/2 and white‐light treated groups exhibited a significant reduction in tumor volume, suggesting that Ir‐MT1/2 activated the anti‐tumor immune response in vivo (Figure 5b,e). DCs, crucial roles in antigen presentation, differentiate into mature DCs (CD86^+^ CD80^+^ DCs) upon antigen stimulation, which in turn activate CD8^+^ T cells and promote anti‐tumor immunity. To further assess the immune activation potential of Ir‐MT1/2, we analyzed the abundance of CD86^+^ CD80^+^ DCs and CD8^+^ T cells in distant tumor tissues by flow cytometry. The results revealed that CD86^+^ CD80^+^ DCs increased by 2.60‐ and 2.52‐fold, and CD8^+^ T cells increased by 3.55‐ and 3.42‐fold in the Ir‐MT1/2 and white‐light treated groups respectively (Figure 5f–i). Additionally, immunohistochemical analysis of primary tumors revealed significant increases in the expression of cleaved caspase‐1 and phosphorylated MLKL in the Ir‐MT1/2 and white‐light treated groups, indicating the induction of pyroptosis and necroptosis by Ir‐MT1/2 (Figure 5j, Supplementary Figures S6 and S7). Immunofluorescence results from primary tumors exhibited a significant decrease in HMGB1 fluorescence intensity and a marked increase in CRT fluorescence intensity, suggesting that Ir‐MT1/2 treatment enhanced the immunogenicity of tumor cells (Figure 5J). Taken together, these results demonstrate that Ir‐MT1/2 induces pyroptosis and necroptosis in vivo, enhancing antigen exposure and activating anti‐tumor immunity.

Despite the promising therapeutic efficacy and favorable biosafety observed in this study, several critical challenges remain for future clinical translation. Firstly, the limited tissue penetration depth of light may limit the efficacy of PDT in deeply located renal tumors. Secondly, the pharmacokinetics, biodistribution, and long‐term clearance of iridium(III) complexes require further systematic investigation to fully evaluate the potential accumulation and off‐target toxicities. In addition, although the current findings demonstrate the significant anti‐tumor efficacy in preclinical models, tumor heterogeneity and the complexity of human immune microenvironment may influence therapeutic outcomes in clinical applications. Therefore, optimization of delivery strategies together with systematic assessment of biosafety will be important steps toward future clinical translation.

## 4 CONCLUSION

In summary, we developed two mitochondria‐targeted iridium(III) photosensitizers, Ir‐MT1 and Ir‐MT2, for synergistic photoimmunotherapy of RCC. By simultaneously inducing pyroptosis and necroptosis, Ir‐MT1/2 enhanced ICD and established a synergistic multi‐network therapeutic framework against RCC. Mechanistically, mtDNA leakage induced by Ir‐MT1/2 activated the cGAS–STING pathway and caspase‐1‐mediated pyroptosis, whereas Ca^2+^ efflux promoted RIPK1/RIPK3 phosphorylation and necroptosis. These death signals collectively promoted tumor cell death and antigen exposure, activating anti‐tumor immune responses in vivo. Overall, this study provides the first evidence that a single iridium complex can induce multi‐RCD cascades to overcome the intrinsic drug resistance of RCC, achieving efficient tumor eradication and potent immune responses, and offering a promising strategy for systemic therapy of RCC.

## CONFLICT OF INTEREST STATEMENT

The authors declare no conflict of interests.

## ETHICS STATEMENT

Experimental animal procedures were approved by the Animal Care and Experiment Committee of Qilu Hospital affiliated to Shandong University (Ethics Code: DWLL‐2024‐330).

## PERMISSION TO REPRODUCE MATERIAL FROM OTHER SOURCES

All data in this study are original and have not been copied from other sources.

## Supporting information

Supporting Information S1

## Data Availability

The data that support the findings of this study are available from the corresponding author upon reasonable request.
