# Supplementary material for: Photoactivated iridium(III) complexes drive pyroptosis–necroptosis synergy for multi‐network photoimmunotherapy of renal cell carcinoma
Source: Smart Mol. 2026 Jul 28:e70087. Online ahead of print. doi: 10.1002/smo2.70087 (PMC13410811; doi:10.1002/smo2.70087)
Supplement: Supplementary file 1 — Supporting Information S1 [file SMO2-9999-0-s001.docx]

Supporting Information

**Photoactivated Iridium(III) Complexes Drive Pyroptosis–Necroptosis Synergy for Multi-Network Photoimmunotherapy of Renal Cell Carcinoma**

*Xin Qin^1#^, Meng-Di Chen^2#^, Wenqi Gao^2#^, Yiping Wang^1#^, Lin-Qing Liu^2^, Zhen Teng^2^, Tienan Qi^1^, Qiyuan Wang^1^, Yuxuan Wang^1^, Daoxiang Li^1^, Keqiang Yan^1^, Ling Pan^3^, Zhongwei Zhao^1^, Shuo Zhao^1*^, Kang-Nan Wang^2*^, Shuai Fu^4*^, Nengwang Yu^5, 6*^*


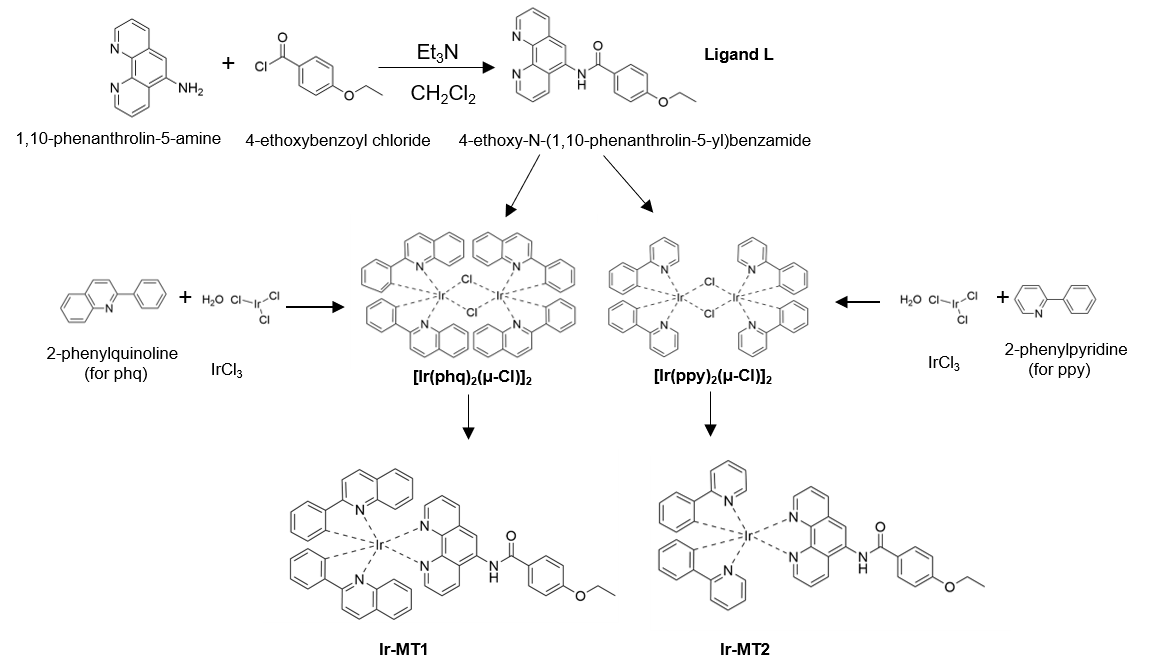


**Scheme S1.** Synthetic procedures of complexes Ir-MT1 and Ir-MT2.

**
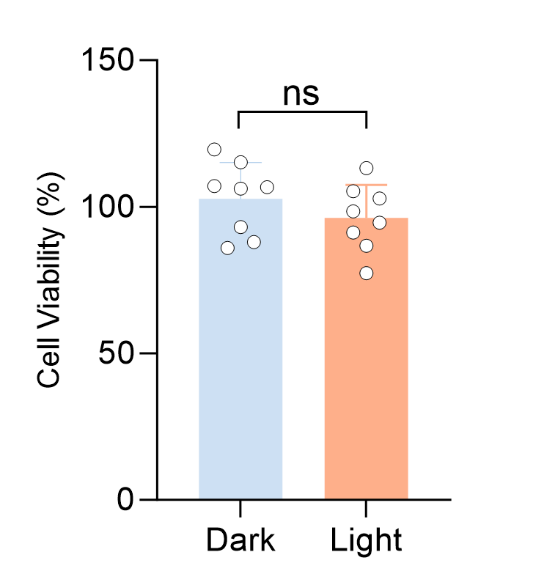
Figure S1.** The cell viability of 786-O cells treated with dark or white light exposure. ns, not significant.

**
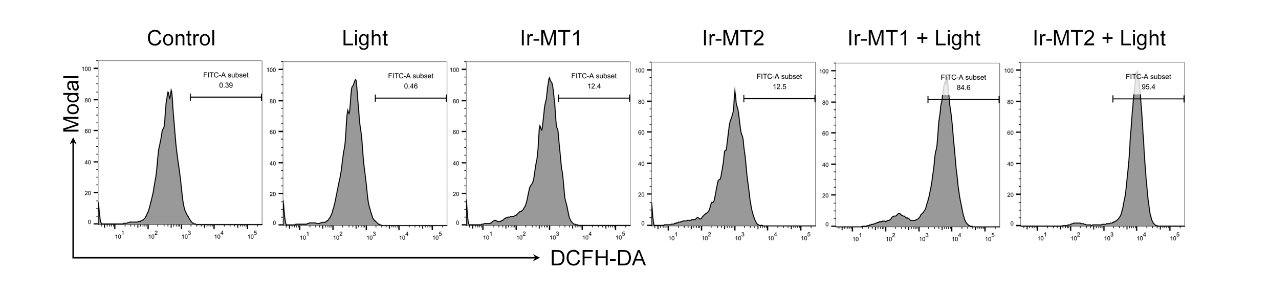
**

**Figure S2.** Intracellular ROS levels in 786-O cells were analyzed by flow cytometry using DCFH-DA staining.

**
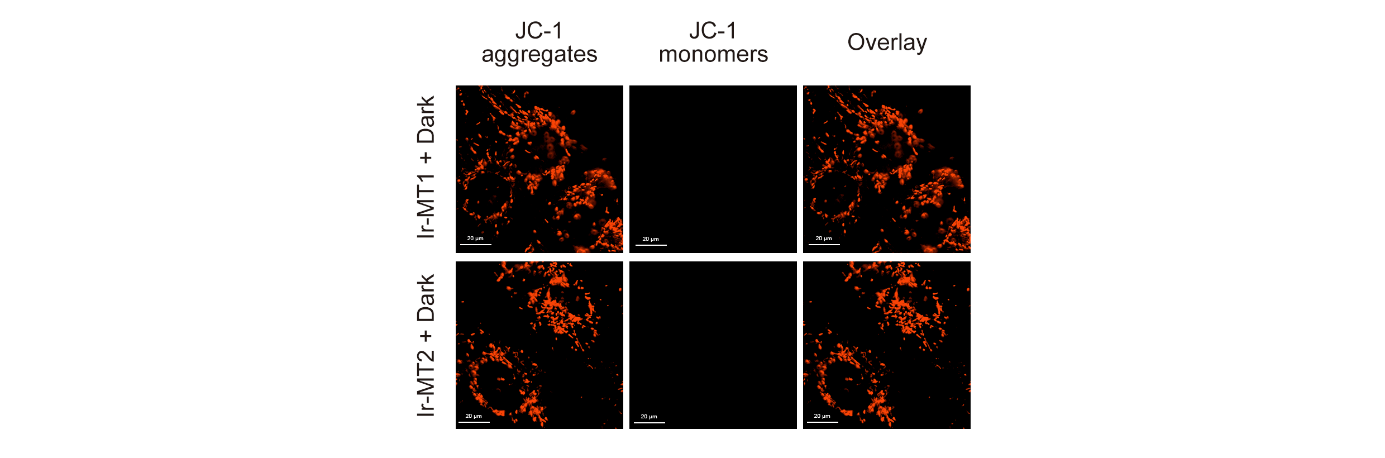
**

**Figure S3.** Mitochondrial membrane potential in 786-O cells was visualized by confocal microscopy using JC-1 staining.

**
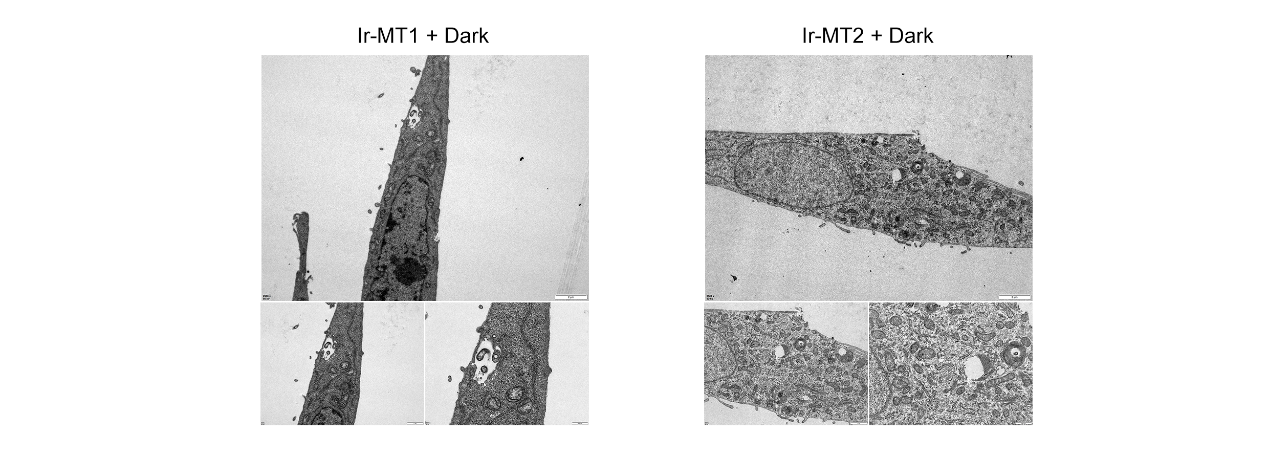
**

**Figure S4.** Morphological changes of 786-O cells induced by Ir-MT1/2 were observed through TEM.

**
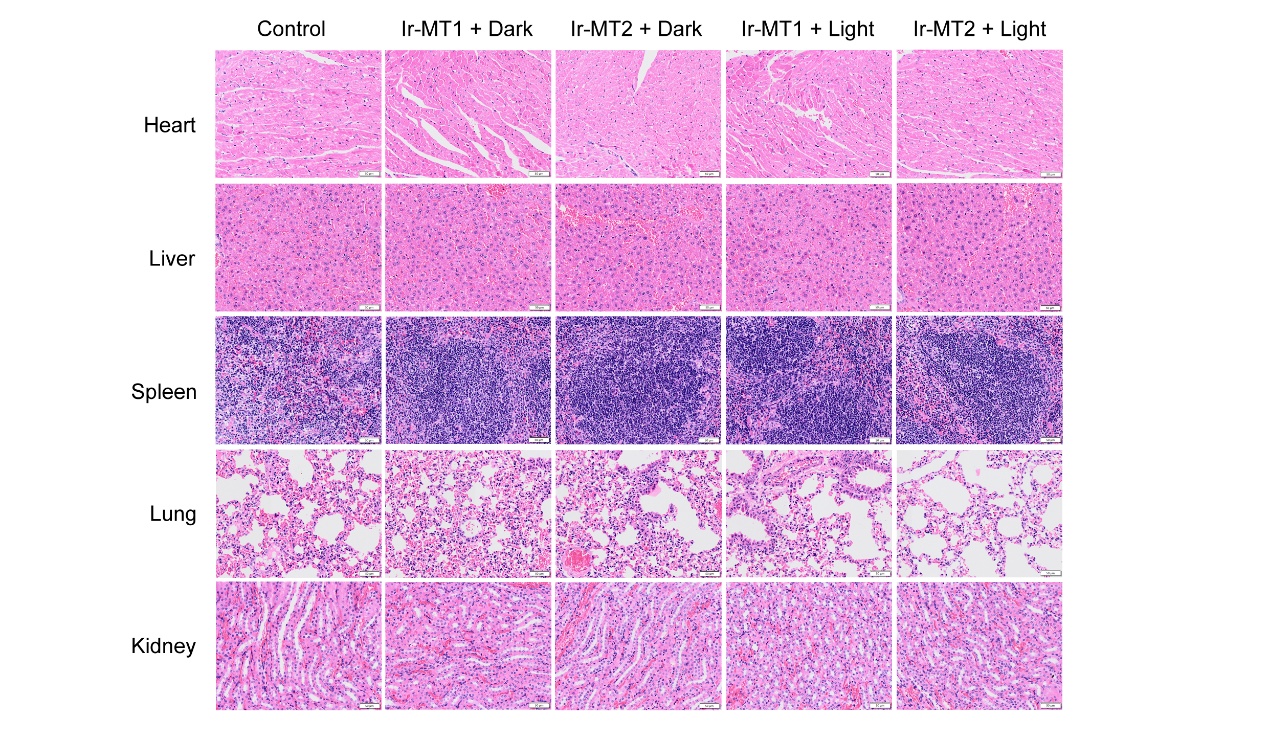
**

**Figure S5.** H&E staining of major organs in BALB/c mice after Ir-MT1/2 treatment

**
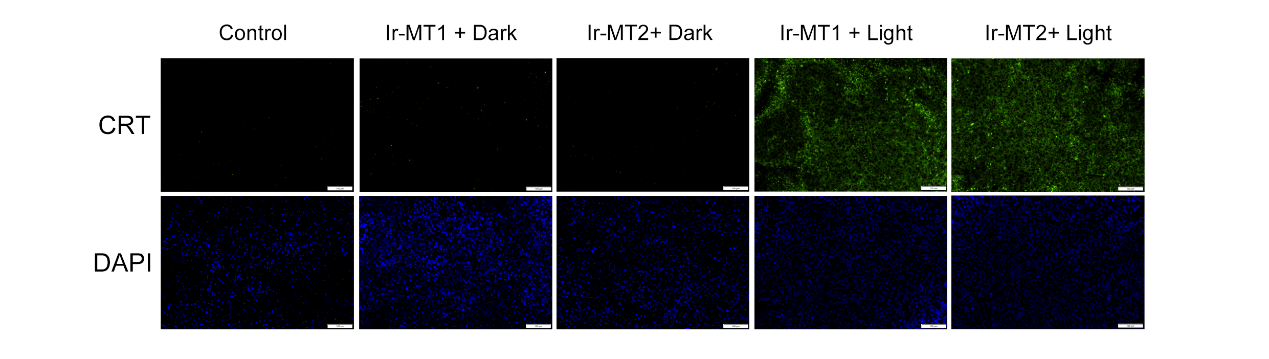
**

**Figure S6.** Immunohistochemical images of CRT in tumor tissues after Ir-MT1/2 treatment.

**
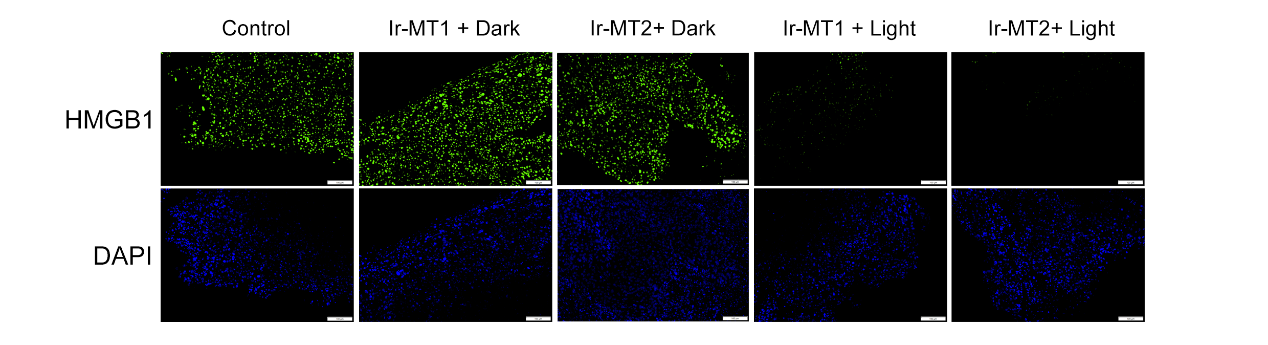
**

**Figure S7.** Immunohistochemical images of HMGB1 in tumor tissues after Ir-MT1/2 treatment.

**Table S1. The photo-physical properties and the lipophilicity (Log P_o/w_) of Ir-MT1/Ir-MT2.**

Data are shown as mean ± SD, n=3.

| Ir-complexes | log P_o/w_^[a]^ | Solvent | λem/nm^[b]^ | τ/ns^[c]^ | ϕPL (%)^[d]^ |
| --- | --- | --- | --- | --- | --- |
| Ir-MT1 |  | 1,4-dioxane | 555 | 547.91±0.4 | 1.01±0.06 |
|  | 1.84±0.05 | DMSO | 553 | 66.26±0.6 | 10.16±0.08 |
|  |  | H_2_O | 558 | 177.07±0.3 | 34.46±0.12 |
| Ir-MT2 |  | 1,4-dioxane | 566 | 163.21±0.4 | 0.25±0.03 |
|  | 2.30±0.07 | DMSO | 567 | 195.12±0.5 | 17.21±0.20 |
|  |  | H_2_O | 565 | 487.08±0.4 | 47.71±0.19 |

^[a]^The log P_o/w_ is the logarithmic ratio of the sample concentration in the organic phase to the aqueous phase.

^[b]^Emission maximum of fluorescence spectra, λ_ex_ = 405 nm.

^[c]^The lifetimes were measured at the emission maxima.

^[d]^The emission quantum yields were determined using [Ru(bpy)_3_]Cl_2_ in 1,4-dioxane, DMSO and H_2_O.

**Table S2. IC_50_ and PI values of Ir-MT1, Ir-MT2 and Cisplatin.**

| Compound | Dark (μM) | Light (μM) | PI* |
| --- | --- | --- | --- |
| **786-O** | | | |
| Ir-MT1 | 5.17 ± 0.33 | 0.24 ± 0.05 | 21.54 |
| Ir-MT2 | 10.72 ± 0.21 | 0.11 ± 0.03 | 97.45 |
| Cisplatin | 63.77 ± 5.70 | 64.27 ± 4.32 | 0.99 |
| **A498** | | | |
| Ir-MT1 | 6.22 ± 0.34 | 0.34 ± 0.08 | 18.29 |
| Ir-MT2 | 13.46 ± 0.44 | 0.16 ± 0.05 | 84.13 |
| Cisplatin | 44.60 ± 2.33 | 44.37 ± 2.01 | 1.01 |
| **Renca** | | | |
| Ir-MT1 | 10.57 ± 1.22 | 0.29 ± 0.02 | 36.45 |
| Ir-MT2 | 8.50 ± 0.25 | 0.17 ± 0.06 | 50 |
| Cisplatin | 57.10 ± 3.80 | 54.30 ± 3.77 | 1.05 |
| **HK-2** | | | |
| Ir-MT1 | 3.29 ± 0.24 | 0.78 ± 0.18 | 4.22 |
| Ir-MT2 | 4.8 ± 0.50 | 0.55 ± 0.14 | 8.73 |
| Cisplatin | 37.97 ± 3.76 | 40.80 ± 4.29 | 0.93 |

*The phototoxicity index (PI) = IC_50_ value (Dark) / IC_50_ value (Light)

**Table S3. Primers used in this study.**

| Species | Gene Symbol | | Sequence (5’→3’) |  |
| --- | --- | --- | --- | --- |
|  |  | | Forward | Reverse |
| Human | | 18s | TAGAGGGACAAGTGGCGTTC | CGCTGAGCCAGTCAGTGT |
| Human | | ND1 | TAACGCACTCTCCCCTGAAC | GTAGCGGAATCGGGGGTATG |
| Human | | ND2 | AGCACCACGACCCTACTACT | CATTTGGGCAAAAAGCCGGT |
| Human | | COX1 | CCCCGATGCATACACCACAT | TCGAAGCGAAGGCTTCTCAA |
| Human | | ATP6 | GAAGCGCCACCCTAGCAATA | GCTTGGATTAAGGCGACAGC |

**Table S4. Antibodies used in this study.**

| Immunogen | Source | Dilution | Vendor | Catalog No |
| --- | --- | --- | --- | --- |
| cGAS | Mouse | 1/1000 | UpingBio | YP-mAb-17228 |
| p-STING | Mouse | 1/1000 | UpingBio | YP-mAb-10413 |
| STING | Mouse | 1/1000 | UpingBio | YP-mAb-17777 |
| p-IRF3 | Rabbit | 1/1000 | UpingBio | YP-Ab-01386 |
| GAPDH | Mouse | 1/5000 | proteintech | 60004-1-Ig |
| NLRP3 | Mouse | 1/1000 | UpingBio | YP-mAb-17806 |
| Caspase-1 | Rabbit | 1/1000 | Abmart | TA5418S |
| Cleaved-Caspase-1 | Mouse | 1/1000 | UpingBio | YP-mAb-00001 |
| GSDMD | Rabbit | 1/1000 | Abmart | TA4012S |
| GSDMD-N | Mouse | 1/1000 | UpingBio | YP-mAb-18001 |
| p-RIPK1 | Mouse | 1/1000 | UpingBio | YP-Ab-18129 |
| RIPK1 | Mouse | 1/1000 | UpingBio | YP-mAb-06716 |
| p-RIPK3 | Mouse | 1/1000 | UpingBio | YP-Ab-17820 |
| RIPK3 | Mouse | 1/1000 | UpingBio | YP-mAb-06748 |
| p-MLKL | Mouse | 1/1000 | UpingBio | YP-Ab-10482 |
| MLKL | Mouse | 1/1000 | UpingBio | YP-mAb-14857 |
| HMGB1 | Rabbit | 1/200 | UpingBio | YP-Ab-02250 |
| CRT | Rabbit | 1/200 | UpingBio | YP-rAb-18027 |
